# Coverage and effectiveness of hypertension screening in different altitudes of Tibet autonomous region

**DOI:** 10.1186/s12889-020-09858-0

**Published:** 2021-01-06

**Authors:** Ci Song, Virasakdi Chongsuvivatwong, Suolang Wangdui, Danzeng Mima, Cuoji Zhuoma, D. Ji, Ouzhu Luobu, Hutcha Sriplung

**Affiliations:** 1 Medical College, Tibet University, Lhasa, 850002 China; 2 Epidemiology Unit, Faculty of Medicine, Prince of Songkla University, Hat Yai, Songkhla, 90110 Thailand; 3 Bomi county centers for disease control and prevention, Nyingchi, 860300 China; 4 Dagze district centers for disease control and prevention, Lhasa, 850100 China; 5 Nagarze county centers for disease control and prevention, Lhokha, 851100 China

**Keywords:** Hypertension screening, Program coverage, Altitude, Tibet

## Abstract

**Background:**

Tibet is an autonomous region in China located around an average altitude of 4500 m above sea level. Since 2012 the local government of Tibet has been providing free physical examinations, including screening for hypertension. However, the coverage and effectiveness of this free program have not been uncovered. This study aims to assess the coverage and effectiveness of hypertension screening and management program in 3 altitude levels of Tibet, and also the determinants of the success of the screening program.

**Methods:**

A stratified cluster survey was conducted among 1636 residents aged 18 years or over in three different altitude areas in Tibet. We adjusted for age and sex based on national census data and used weighted logistic regression models to find factors associated with hypertension screening.

**Results:**

The coverage of the hypertension screening program evaluated by participation rate in the previous screening was 94.9%, while 24.7% (95% CI: 22.1–27.3%) of them were diagnosed with hypertension. Females and alcohol drinkers were more likely to be screened. Among those diagnosed with hypertension, 28.7% had it under control. High altitude areas had a high proportion of controlled hypertension. The overall rate of controlled hypertension in high, moderate and low altitude areas was 35.1% (95% CI: 24.8–45.3%), 32.7% (95% CI: 22.2–43.2%) and 23.7% (95% CI: 14.7–32.6%), respectively. Younger aged persons were more likely to have better control of their hypertension.

**Conclusions:**

The coverage of hypertension screening in Tibet was high, especially in the low altitude areas. However, the effectiveness of hypertension control was low, indicating a need to implement the treatment adherence routines into the current screening interventions.

**Supplementary Information:**

The online version contains supplementary material available at 10.1186/s12889-020-09858-0.

## Background

Hypertension (HT) is a significant health problem affecting 31.1% of the global population and leads to morbidity and mortality worldwide [1]. High blood pressure (BP) is a prevalent and essential promoter of vascular damage resulting in cardiovascular diseases (CVD). Many risk factors for hypertension are behavioral and modifiable. The control of blood pressure is crucial in the prevention of CVD [2, 3].

China initiated the community-based prevention of CVD project in 1969 as a comprehensive intervention program that focused on the prevention and treatment of hypertension. Since the 1970s, similar centers and pilot programs expanded to various provinces and autonomous regions of China [4]. According to the results of past national hypertension screening surveys, the prevalence of hypertension among the general population aged 18 years or older in China increased from 7.7% in 1979 to 18.0% in 2002 and 38.0% in 2010 [5–7] In 2009, the fourth new medical reform policies brought community-based prevention of hypertension and diabetes into the agenda of national public health services [8, 9]. For several decades, the intervention program has extended from the treatment of individuals with hypertension to large-scale management in communities and the prevention of comorbidities [4].

Tibet is an autonomous region in the southwest of China located around an average altitude of 4500 m above sea level [10]. In 2015 the estimated population of Tibet was 3.23 million, with ethnic Tibetans comprising 90% of the whole community [11]. In 2015, the regional’s disease surveillances showed three major diseases in Tibet that were chronic diseases, infectious diseases and maternal nutritional deficiency diseases and top five causes of death that were cerebrovascular diseases, respiratory diseases, heart diseases, digestive system diseases and malignant tumors [12]. In Tibet, the criteria for hypertension diagnosis is based on the 2018 Chinese guidelines for prevention and treatment of hypertension. The prevalence of hypertension in Tibet (55.9%) is higher than the Chinese national level (29.6%), and is the highest among all provinces [13, 14]. Moreover, a study reported that the rate of CVD-related morbidity and mortality was higher than in other provinces of China [15]. Our previous survey showed that the prevalence of HT decreased with increasing elevation and increased with advanced age and increasing BMI value [16]. Based on previous studies, the association between altitude and hypertension prevalence is controversial [10, 17] Mingji et al. [10] found that geographic and socioeconomic status had significant effects on the awareness and subsequent treatment and control of hypertension among people living in different altitude areas. The local government of Tibet has been sponsoring free physical examinations for herder-farmers since 2012 [18]. In 2017, more than 3 million urban and rural residents had their health details recorded in the government health information system, equating to coverage of more than 97% in Tibet [19].

The physical examination program included simple measurements such as height, weight, and blood pressure measurement, and also the sophisticated tests such as vision, blood examination, and echocardiography. Those diagnosed with hypertension receive lifestyle intervention and medical treatment according to the national guidelines [20], and the hypertension information system secured their records. Individuals classified as high risk of hypertension or pre-hypertension are managed promptly at the nearby health center. Health personnel at the local health care center follow hypertensive patients up every three months to check their blood pressure and monitor any complications and side effects of treatment [21]. Patients are transferred to the upper level of medical care if needed.

The researchers conducted two parallel surveys to find the prevalence of hypertension in relation to the 3 levels of geographical altitude in Tibet and to document the epidemiological risk factors for HT, both general and specific to Tibetan people. It was published earlier in February 2020 [16], Another survey, the current report, tried to estimate the coverage of the existing HT screening program provided by the government of Tibet Autonomous Region which required people to come to the health service stations using the local administrative network down to the villages.

Although the administrative data is present for the whole region, information on screening in different altitude areas was lacking. At this moment, there has been no research reporting the coverage of the target population, i.e. the number and percentage of people screened for hypertension in Tibet. As transportation barriers and high altitudes in Tibet complicates the control of hypertension, the evaluation of the program in this area needs further scrutiny. This study aims to assess the coverage and effectiveness of hypertension screening and management in different altitude areas of Tibet.

## Materials and methods

### Study design

We conducted a stratified cluster survey from September to December 2017 to determine the coverage and effectiveness of hypertension screening and management program in 3 altitude levels of Tibet, and also the determinants of the success of the screening program.

### Study setting

This study was combined within our previous report on the relationship of hypertension prevalence and geographic altitude in Tibet [16]. Three different altitude areas were purposively selected: Bomi county of Nyingchi city, Dagze district of Lhasa city, and Nagarze county of Lhokha city. These three areas had an average altitude of 2500 m, 4100 m, and 4500 m above sea level, respectively [22]. Bomi County is the farthest from the capital city (Lhasa), followed by Nagarze county and Dagze District, with average distances from the capital city of 630, 127, and 50 km, respectively. In Bomi County, the primary sources of income are agriculture, forestry, and tourism. Being close to the capital city of Lhasa, Dageze district is more urbanized, although, in some parts, agriculture is the primary source of income. Nagarze county is situated in south-eastern Tibet, mostly surrounded by hills where animal husbandry has become the primary source of income for residents.

### Sample selection

Two townships located within 50 km from the center of each county were selected using simple random sampling. Thus, we chose a total of 6 townships as the primary sampling units. The most recent prevalence (p) of hypertension in Lhasa city was 51.2% [23]. With a two-sided, 95% confidence interval, an error (d) of 0.05, a design effect (*deff*) of 1.3, and including 10% non-respondents, we required a total of 550 participants in each county by using the following formula. We randomly selected eligible participants from each county. The formula for sample size calculation to get an adequate prevalence of hypertension was:

$$ n={Z}_{1-\frac{\propto }{2}}^2\ p\frac{1-p}{d^2}, $$

*n*_*Adjust*_ *= n x deff*.

We ran a multistage cluster sampling by randomly selecting 10 administrative villages from 6 townships in the three study areas. The researcher team went to the villages and did participant invitation through the appointment of the head of the village.

### Inclusion and exclusion criteria

Eligibility criteria for participants included age ≥ 18 years, Tibetan ethnicity, and residents in the village for at least one year. Excluded from this survey were those who had severe mental dysfunction, pregnancy, or severe complications of hypertension.

### Data collection

The researchers invited eligible participants to the nearest local primary health center or village committee offices. After giving informed consent, all study participants were physically examined by trained investigators following standard protocols. Physical measurements included weight, height, and blood pressure. The body weight and height of participants wearing no shoes or overcoat were measured using the Suhong RGZ-120 height and weight scale. Before the first examination of their blood pressure, we allowed participants to relax for at least five minutes in a quiet room. Investigators advised all participants to avoid drinking tea and alcohol, cigarette smoking, over-exercising, and to void urine half an hour before their examination. In this survey, we used an electronic sphygmomanometer with high reliability and validity (Omron HEM-7201 automatic blood pressure monitor) at a high altitude area [24]. Blood pressure was measured twice at 60 s intervals for all participants. Those who had a discordant blood pressure of greater than ten mmHg on the previous two measurements had a third measurement taken. The final result was the arithmetic mean of all BP measurements. To explore the participants previously diagnosed with hypertension, we administered self-completed questionnaires and confirmed the results by checking the record books of the participants. The questionnaire and case record form (Supplementary Table 1) used in this study were developed by the researcher team and used in our previous survey in estimating the prevalence of HT in Tibet reported separately [16].

### Variable definitions

The outcome variables are subjects who were screened for hypertension and among the known hypertension cases, those who were controlled across the different altitude areas in Tibet. Independent variables included socio-demographic characteristics, biological, and behavioral determinants, and history of diseases. Behavioral determinants included the consumption of tobacco and alcohol, and biological factors included body mass index (BMI) defined as the weight (kg) divided by the square of height (m^2^) and waist circumference. According to Chinese BMI classification, the BMI range for overweight is from 24.0–27.9 kg/m^2^, and the cut point for obesity is ≥28.0 kg/m^2^ [25].

We identified those who had regular measurements by community health workers in the hypertension intervention program to determine the screening coverage of hypertension screening. We recorded the results of the routine hypertension screening as hypertension or no hypertension detected by the routine screening by community health workers.

We defined the effectiveness of hypertension screening in terms of retention in care after diagnosis and initiation of treatment [26] as the proportion of those previously diagnosed hypertension by local health workers who had systolic blood pressure (SBP) < 140 mmHg and diastolic blood pressure (DBP) < 90 mmHg.

Individuals previously informed by a doctor or local health worker about their hypertension status, despite their current hypertension status, were considered to have hypertension awareness. Individuals who responded “no” to the question: “In the past, have you received measurement of hypertension by a local doctor or healthcare provider?” and had hypertension during their physical examination, were categorized as unaware hypertension.

The presence of hypertension was defined as SBP ≥ 140 mmHg and/or DBP ≥ 90 mmHg, and/or self-reported treatment for hypertension with antihypertensive medications taken in the past two weeks [27].

Participants who answered “yes” to the question “In the past, have you received a diagnosis of hypertension by a local doctor or healthcare provider?” were categorized as previously diagnosed hypertension.

### Statistical analysis

We described the characteristics of the participants in our study by a weighted analysis. We observed the difference in the distribution of age and gender between the study participants and the list of residents in the villages (Supplementary Table 2). We used the survey design to adjust the estimates, and the iterative proportional fitting (raking) to reduce the sampling bias by fitting the data using known demographic characteristics from the 2010 census. We used R version 3.5.1 (https://cran.r-project.org) to analyze the data. We used the survey-weighted logistic regression models to find factors associated with the screening of hypertension in the past.

A Venn diagram visualizes the overlapping sets of newly discovered hypertension cases by this study, previously diagnosed hypertension by screening program, and those who received antihypertensive treatment in the past, both controlled and uncontrolled states.

Figure . 1 schematizes the three groups of people. Those who were covered by this survey are shown in the dotted-lined circle. The solid-lined circle represents those who were previously approached by the screening program. The small dash-dot-lined circle represents those who were screened and received antihypertensive treatment. The segment marked “a” represents those who diagnosed with hypertension but never tested or unscreened hypertension in the past and denoted as unaware hypertension. Uncontrolled hypertension (or diagnosed hypertension in both the survey and screening program) is the summation of segments “b” and “d.” Segment “c” represents those who previously had a history of diagnosed hypertension but normal blood pressure in our study. Segments “d” and “e” represent those uncontrolled and controlled hypertensive patients after medical treatment, respectively. Finally, anyone falling outside the circles marked as “f” is normotensive or non-hypertensive subjects.

**Fig. 1:**
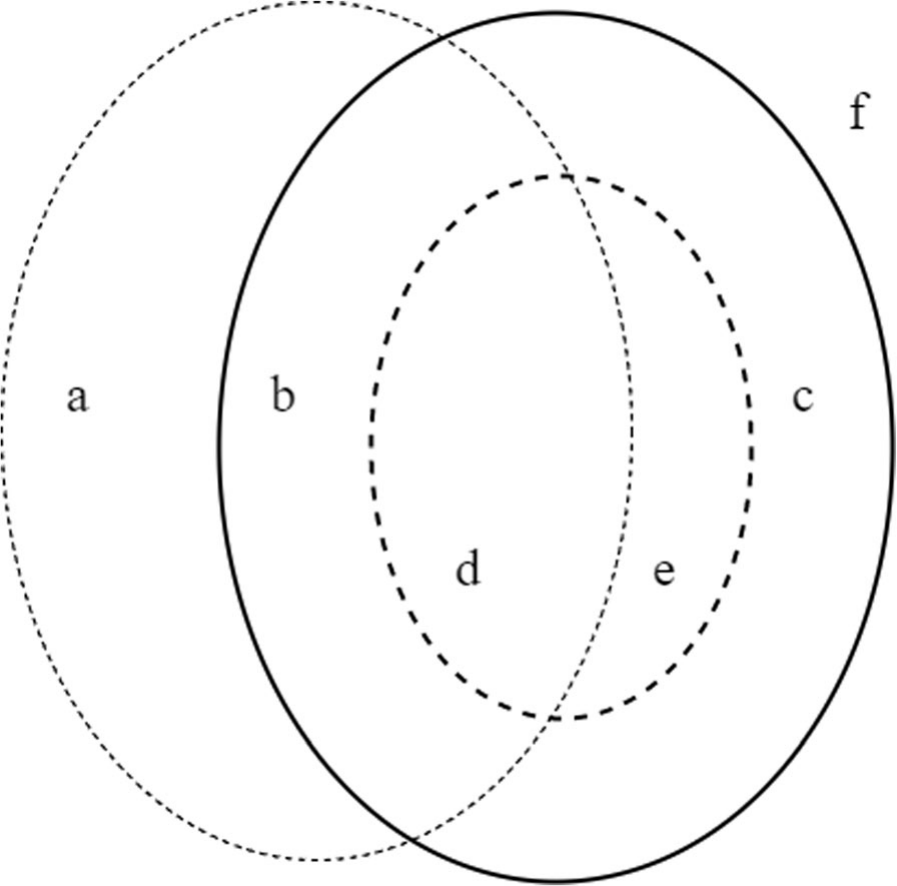
Venn diagram showing the sets of participants and their status of previous hypertension diagnosis, treatment, and hypertension status at the survey

The effective coverage was defined by Shengelia et al. (2005) [28] and later by Ng et al. (2014) [29], as the fraction of potential health gain that was actually delivered to the population through the health system. The calculation of the effective coverage follows the following formula:

*EC*_*ij =*_ *U*_ij*_Q_*ij*_*/N*_*ij*_*.*

Where subscriptions *i* and *j* represent individual and intervention. *EC*_*ij*_ is the effective coverage of individual *i* with intervention *j*. In our case, where the intervention is only the HT screening program, thus, *i* remains in the formula, and *j* is omitted. Then,

*EC*_*i* =_ *U*_i*_Q_*i*_*/N*_*i*_, or *EC* = U_*_Q/N, in a simple term.

Where *Q* is the quality or health gain ratio. *U* is the utilization of health service and refers to the probability that the individual with a need will receive the intervention. *N* is the need indicator, which refers to individuals who will gain actual benefits from receiving or true need; if *N = 1* is the true need for receiving the healthcare services and *N = 0* for individual no need for coverage. In this survey, we calculated the effective coverage of HT screening by replacing *U* with the coverage of HT screening program, and *Q/N* is the effectiveness of HT treatment, both by medical and lifestyle modification, among the screened population.

Variables excluded from the final multivariate model were those with a univariate *p*-value greater than 0.2. Factors assisting control of hypertension programs were identified using survey-weighted logistic regression modeling and determined based on a backward step-wise process and included only variables with a p-value less than 0.05.

## Results

Among a total of 1636 study participants, 1020 (62.3%) were female. The un-weighted characteristics of participants by the status of hypertension screening in the past are shown in Supplementary Table 2. Local health workers screened a total of 1539 (94.1%) participants from the screening program, of which 37.4% were diagnosed with hypertension.

Table 1 shows the weighted results applied to the whole population. Almost 95% of residents were previously screened for hypertension by a hypertension intervention program or free physical examination project, especially in the low altitude area, above 97% of the participants were screened. Gender, age, occupation, alcohol use, and altitude were factors significantly associated with prior screening status. Among those prior screened, 24.7% (95% CI: 22.1–27.3%) were diagnosed with hypertension. Hypertension status significantly associated with gender, age group, marital status, education, BMI level, and altitude.

**Table 1:** Characteristics of the participants by the screening of hypertension in the past, in estimated number of Tibetan people and percentage and 95% confidence interval

	History of hypertension screening									***p*** - value
	Screened							Not screened		
	Hypertension									
**Characteristics**	**Total**		**Yes**		**No**					
	Est. N	% (95% CI)	Est. N	% (95% CI)	Est. N	% (95% CI)	***p*** **- value**	Est. N	% (95% CI)	
Overall	64,227	94.9 (93.2–96.6)	15,881	24.7 (22.1–27.3)	48,346	75.3 (72.7–77.9)		3426	5.1 (3.4–6.8)	
Gender							0.003			0.027
Male	32,613	93.4 (90.3–96.4)	9835	30.2 (25.1–35.2)	22,778	69.8 (64.8–74.9)		2309	6.6 (3.4–9.7)	
Female	31,613	96.6 (95.3–97.9)	6045	19.1 (15.4–22.9)	25,567	80.9 (77.1–84.6)		1116	3.4 (2.1–4.7)	
Age group							< 0.001			0.040
18–39	38,415	96.8 (94.2–99.4)	4393	11.4 (7.6–15.3)	34,022	88.6 (84.7–92.4)		1275	3.2 (0.6–5.8)	
40–59	19,226	92.3 (90.2–94.4)	7013	36.5 (32.9–40.1)	12,212	63.5 (59.9–67.1)		1598	7.7 (5.6–9.7)	
60 +	6585	92.3 (88.7–95.8)	4473	67.9 (61.7–74.2)	2111	32.1 (25.8–38.3)		552	7.7 (4.2–11.3)	
Marital status							< 0.001			0.468
Single/separated	7858	96.9 (92.4–100)	3595	45.8 (36.0–55.5)	4262	54.2 (44.5–64.0)		252	3.1 (0–7.0)	
Married	56,368	94.7 (92.8–96.5)	12,284	21.8 (19.1–24.5)	44,083	78.2 (75.5–80.9)		3175	5.3 (3.5–7.1)	
Education							0.031			0.319
None	30,351	96.3 (95.1–97.4)	8630	28.4 (24.7–32.2)	21,720	71.6 (67.8–75.3)		187	3.7 (2.5–4.9)	
Primary school	26,191	93.1 (89.8–96.4)	6210	23.7 (19.2–28.2)	19,981	76.3 (71.8–80.8)		487	6.9 (3.5–10.2)	
Middle school and above	7683	96.1 (89.4–100)	1040	13.5 (4.4–22.6)	6643	86.4 (77.4–95.6)		279	3.9 (0.0–10.6)	
Occupation							0.096			0.020
Agriculturalist	55,782	96.1 (94.8–97.4)	13,343	23.9 (21.2–26.6)	42,438	76.1 (73.4–78.8)		2240	3.9 (2.6–5.2)	
Herdsman	4294	87.4 (76.5–98.4)	1656	38.6 (24.7–52.5)	2638	61.4 (47.5–75.3)		617	12.5 (1.6–23.5)	
Other	4149	87.9 (74.8–100)	881	21.2 (7.6–34.9)	3269	78.8 (65.1–92.4)		569	12.1 (0--25.2)	
Income (CNY),							0.283			0.760
≤ 2500	54,426	95.0 (93.3–96.7)	13,879	25.5 (22.7–28.3)	40,546	74.5 (71.7–77.3)		2.863	5.0 (3.3–6.7)	
2501–5000	7906	93.9 (87.3–100)	1767	22.3 (13.3–31.4)	6139	77.7 (68.6–86.7)		513	6.1 (0–12.6)	
≥ 5001	1894	97.4 (92.3–100)	235	12.4 (1.0–23.8)	1659	87.7 (76.2–99.0)		49	2.6 (0–7.6)	
Tobacco use							0.902			0.325
Yes	17,447	93.4 (89.2–97.8)	4260	24.4 (18.8–30.0)	13,187	75.6 (70.0–81.2)		1224	6.5 (2.3–10.8)	
No	46,779	95.5 (93.8–97.1)	11,620	24.8 (21.7–28.0)	35,159	75.2 (72.0–78.3)		2202	4.5 (2.8–6.1)	
Alcohol use							0.693			0.013
Yes	17,133	97.5 (96.0–99.0)	4048	23.6 (17.0–30.2)	13,085	76.4 (69.7–83.0)		443	2.5 (1.0–4.0)	
No	47,093	94.0 (81.7–96.3)	11,833	25.1 (22.3–28.0)	35,260	74.9 (72.4–77.7)		2983	6.0 (3.7–8.3)	
BMI (kg/m^2^)							< 0.001			0.112
≤ 23.9 (Normal)	37,148	96.2 (94.4–98.1)	6031	16.2 (13.6–18.9)	31,117	83.8 (81.1–86.4)		1459	3.7 (1.9–5.6)	
24–27.9 (Overweight)	18,494	94.4 (91.3–97.6)	5609	30.3 (24.8–35.9)	12,885	69.7 (64.1–75.2)		1095	5.6 (2.4–8.7)	
≥ 28.0 (Obese)	8583	90.8 (84.0–97.6)	4240	49.4 (39.2–59.6)	4343	50.6 (40.4–60.8)		871	9.2 (2.4–16.0)	
Altitude level (m)							< 0.001			0.002
Low (2500–3499)	24,892	97.7 (96.0–99.4)	8405	33.8 (28.5–39.0)	16,486	66.2 (61.0–71.5)		585	2.3 (0.6–4.0)	
Middle (3500–4399)	10,082	96.4 (94.9–97.9)	2393	23.7 (18.7–28.8)	7689	76.3 (71.2–81.3)		378	3.6 (2.1–5.1)	
High (4400–5300)	29,252	92.2 (89.0–95.5)	5083	17.4 (13.7–21.0)	24,169	82.6 (79.0–86.3)		2463	7.8 (4.5–11.0)	

*Note*: data are presented as the mean percentage with 95% CI; the Chi-square test was used for categorical variables

*Abbreviations*: *CI* confidence interval, *CNY* Chinese yuan, *BMI* body mass index, *Est. N* estimated number

Table 2 summarizes the risk factors of previous screening for hypertension from the multivariate analysis. Gender, alcohol consumption, and living altitude were significantly associated with the last screening for hypertension, after adjustment for other potential confounding factors. Females and alcohol drinkers were more likely to be screened while the proportion of the population screened declined with increasing altitude.

**Table 2:** Factors associated with previous screening for hypertension

Variable			*P-value
	Percent Screened (95% CI)	Odds ratio (95% CI)	
Total	94.9 (93.2–96.6)		
Gender			
Female	96.6 (95.3–97.9)	Ref.	0.034
Male	93.4 (90.3–96.4)	0.45 (0.21–0.95)	
Age group			0.143
18–39	96.7 (94.2–99.4)	Ref.	
40–59	92.3 (90.3–94.4)	0.49 (0.20–1.24)	
60 +	92.3 (88.7–95.8)	0.47 (0.17–1.32)	
Occupation			0.335
Agriculturalist	96.1 (94.8–97.4)	Ref.	
Herdsman	87.4 (76.5–98.4)	0.57 (0.19–1.74)	
Other	87.9 (74.8–100.0)	0.40 (0.10–1.57)	
Alcohol use			0.004
No	94.0 (81.7–96.3)	Ref.	
Yes	97.5 (96.0–99.0)	3.39 (1.36–8. 45)	
BMI (kg/m^2^)			0.055
≤ 23.9 (Normal)	96.2 (94.4–98.1)	Ref.	
24–27.9 (Overweight)	94.4 (91.3–97.6)	0.58 (0.27–1.25)	
≥ 28.0 (Obese)	90.8 (84.0–97.7)	0.29 (0.10–0.87)	
Altitudes level (m)			0.010
Low (2500–3499)	97.7 (96.0–99.4)	Ref.	
Middle (3500–4399)	96.4 (94.9–97.9)	0.59 (0.26–1.36)	
High (4400–5300)	92.2 (89.0–95.5)	0.28 (0.10–0.77)	

*Note*: Ref.: the reference group of each predictor. * Likelihood ratio test

*Abbreviations*: *CI* confidence interval, *BMI* body mass index

Figure 1 and Table 3 illustrate the proportions of participants with current hypertension and previous hypertensive diagnosis and treatment. The overall unaware hypertension rate was 12.6%, and as shown in Table 3, the highest rate occurred in the middle altitude area (16.9%). The overall uncontrolled hypertension rate was 71.3%, and the low altitude area had the highest rate.

**Table 3:** Hypertension awareness and control status in the Tibetan population stratified by altitude

Outcome variable	Total		Altitude						*P*-value
			Low (2500–3499 m)		Middle (3500–4399 m)		High (4400–5300 m)		
	N	% (95%CI)	N	% (95%CI)	N	% (95%CI)	N	% (95%CI)	
Awareness rate of hypertension among those discovered in this study									0.045*
Unaware (a)	8554	12.6 (10.2–15.1)	3757	**14.7 (10.2–19.3)**	1767	16.9 (12.5–21.3)	3030	9.6 (6.0–13.1)	
Aware (b + c + d + e + f)	59,098	87.3 (84.9–89.8)	21,720	85.3 (80.7–89.8)	8693	83.1 (78.7–87.5)	28,684	90.4 (86.9–94.0)	
Hypertension control status among those previously diagnosed with hypertension									0.150
Uncontrolled (b + d)	**11,326**	**71.3 (65.4–77.2)**	6416	76.3 (67.4–85.2)	1610	67.3 (56.8–77.8)	3301	64.9 (54.6–75.2)	
Controlled (c + e)	4554	28.7 (22.8–34.6)	1990	23.7 (14.7–32.6)	783	32.7 (22.2–43.2)	**1782**	**35.1 (24.8–45.3)**	
Hypertension control status among those not prescribed anti-hypertension medication									0.321
Uncontrolled (b)	2471	60.5 (49.0–72.0)	1041	66.7 (48.9–84.5)	494	70.9 (47.5–94.2)	936	51.2 (34.0–68.5)	
Controlled (c)	1614	39.5 (28.0–51.0)	521	33.3 (15.5–51.1)	203	29.1 (5.8–52.5)	**890**	**48.8 (31.5–66.0)**	
Hypertension control status among those who were prescribed anti-hypertension medication									0.302
Uncontrolled (d)	8855	75.1 (68.2–81.9)	5374	78.5 (68.3–88.8)	1116	65.8 (54.6–77.0)	2364	72.6 (61.3–84.0)	
Controlled (e)	2940	24.9 (18.1–31.8)	1469	21.5 (11.2–31.7)	579	34.2 (23.0–45.4)	892	27.4 (16.0–38.7)	
Effective coverage of HT screening		28.7 (22.8–34.6)		43.7 (30.8–56.5)		17.2 (10.1–24.3)		39.1 (27.5–50.8)	0.154

*Note*: The data are weighted percentage with 95% CI shown in brackets; Pearson’s chi-square test with Rao-Scott adjustment was used to compare

In this regard, the overall rate of hypertension controlled by non-medication prescription was 39.5%, and the coverage tended to be better in the high altitude areas with no statistical significance. Among those with medical treatment, the overall controlled hypertensive rate was 24.9%, and the rates were similar in the three altitude levels. In addition, we observe there were differences in the effective coverage of HT screening in different areas. The overall effective coverage of HT screening was at 27.2%. The proportion of effective coverage was slightly higher at high altitude area. There is likely a positive correlation between effective coverage and the effectiveness of HT screening in different areas.

Table 4 shows factors associated with controlled hypertension (overall, by treatment, and by lifestyle modification) among those with previously diagnosed hypertension. Altitude level and age were significantly associated with overall controlled hypertension, while age and gender were significantly related to controlled hypertension by treatment and lifestyle modification, respectively. Those who lived in the middle and high altitudes and were aged 18–39 years were more likely to control their hypertension.

**Table 4:** Factors associated with controlled hypertension among those with previous hypertension

Variables	Overall controlled hypertension		Controlled hypertension by medical treatment		Controlled hypertension by lifestyle modification	
	aOR (95% CI)	P^*^	aOR (95% CI)	P*	aOR (95% CI)	P^*^
Altitude level (m)		0.030		0.080		0.115
Low (2500–3499)	1.00		1.00		1.00	
Middle (3500–4399)	1.98 (1.01–3.90)		2.70 (1.38–5.28)		1.51 (0.34–6.76)	
High (4400–5300)	2.19 (1.08–4.43)		1.80 (0.71–4.59)		2.91 (1.05–8.05)	
Age group (year)		0.002		0.017		0.088
18–39	1.00		1.00		1.00	
40–59	0.34 (0.15–0.78)		0.33 (0.11–0.94)		0.45 (0.15–1.35)	
60 +	0.23 (0.09–0.54)		0.23 (0.08–0.68)		0.25 (0.07–0.89)	
Gender		0.161		–		0.019
Female	1.00		–		1.00	
Male	0.66 (0.37–1.17)		–		0.31 (0.12–0.82)	

*Likelihood ratio test p-value, *aOR* adjusted odds ratio

Hypertension control via drugs was more likely among people aged under 40 years and among those living in middle altitude areas, while hypertensive control via lifestyle modification was more likely among those who lived in high altitudes without statistically significant. Females were also more likely to control their hypertension with lifestyle modification.

## Discussion

The proportion of hypertension screening was highest among Tibetans who lived at low altitudes. Other factors, such as gender, alcohol use, and BMI, also played some role in the coverage of hypertension screening. Around 87% of Tibetans aged over 18 years were screened for hypertension, and 28.7% of those diagnosed with hypertension had it under control. Despite the high screening coverage, the screening program’s effectiveness was considered acceptable at around 30% of controlled hypertension. A higher proportion of hypertension awareness and controlled hypertension was found among those living in high altitude areas. The overall effective coverage of HT screening was only at 27.2%. The program’s effectiveness was dependent on altitude, age, and gender. Younger aged residents, females, and those who lived in the middle and high altitudes tended to have higher control rates.

More than 95% of the participants living at the low and middle altitude areas were previously screened by a hypertension intervention program or free physical examination project, while the screening rate was over 90% for people living at high altitudes. This finding indicates that the coverage of the hypertension screening program was high in all three altitude levels. The fact that townships in the low altitude areas are more developed than in the high areas could explain the higher screening coverage in the low and middle elevation areas where people and health care personnel have been adapting to the Western Development Strategy [30].

High coverage of hypertension screening was more prevalent among females and alcohol drinkers as shown in Table 2. Studies assessing the population coverage of hypertensive screening demonstrated that females had a slightly more significant proportion of hypertension testing than males [31, 32]. We found no effect of age on the screening coverage. One of the reasons was that age was independent of the demand for the health care of individuals. The health care system and its personnel actively ran the program based on the household registration.

In our study, those who drank alcohol tended to be more engaged in the hypertension screening program. The Tibetan government set up the hypertension screening program to find people with high blood pressure. It is relatively easy for local health personnel to target those who drink since they tend to socialize in public places. It is a usual practice of health care personnel to find people with hypertension among vulnerable groups and give them appropriate treatment. As a result, the program may have recruited more drinkers than expected. However, it can result in a bias in estimating the attitude of people to come for screening. We don’t know whether or not drinkers have changed their drinking behavior and reduced the amount of alcohol intake. Alcohol drinking culture is still prevalent in Tibet, for people to show their hospitality and to reduce stress, facilitate social interaction, and foster good interpersonal relationships [33, 34]. So far, there has been no program for alcohol consumption reduction in Tibet. Experiences from European countries showed that even with the existence of screening and appropriate interventions for hazardous alcohol use and use disorders, a lack in implementing the measures prevented success in non-communicable disease control [35].

We found that the hypertension awareness rate was 87.3% (95% CI: 84.9–89.8%), and it was higher than the rate in Lhasa reported in 2013 of 63.5% [36]. Our finding was also higher than the results of other studies conducted in different regions of China [6, 14], and among Canadian adults [37]. Financial affordability acts as a strong barrier compared to physical accessibility and acceptability of the hypertension screening program in some countries [38], but not in Tibet, where hypertension screening has been a part of the universal health benefits package since 2012 [19]. In general, the awareness of health and healthcare utilization is related to distance to and convenience of the health service [28].

The hypertensive awareness rate was the highest at high altitude areas where it was 90.4% (95%CI: 86.9–94.0%). At high elevation in Tibet, people live at a long distance from the health facilities. Those living at high altitudes are likely the target of health researches and various kinds of services by the government and academic institutes, and they are therefore more likely to be approached by the health services for hypertension screening.

The overall control rate of hypertension in our study was 28.7%. It was about three times higher than the finding of 9.6% reported in a study that was conducted in five provinces in southwest China [39]. The overall hypertension control rate increased with increasing altitude. The HT control rate, both on the whole and among those who took medical treatment, was higher in young people than the elderly. Such a better hypertension control among young persons is different from the findings in China [40] and in the United States [41], while the results in Pakistan were similar to our study [42]. In Tibet, health personnel have been actively inviting villagers to participate in hypertension check-up procedures. The study from Pakistan mentioned ‘hypertension screening camps’ which implies the active recruitment of participants. The method used to recruit participants in hypertension screening, therefore, seems to affect the relationship between age and the control rate of hypertension.

The effective coverage of HT screening in this study period was a little over a quarter (27.2%). It is lower than the effective coverage of HT screening in Thailand at 49.9% [31]. The main reason for the low effective coverage was a big portion of those who found HT by screening got insufficient control of HT. We found the effectiveness of hypertension control by lifestyle modification was better in females than males (Table 4). The phenomenon happened in the reports from the United States and Africa [40, 43]. A meta-analysis of studies did not document gender differences in hypertension treatment results [44]. The conclusion was that socioeconomic and cultural factors could explain such differences by gender. Studies in the United States and Africa mentioned a better practice of health-seeking behavior among females for chronic disease than males [32, 45]. Such a better health-consciousness among females may explain the finding that they got better control from lifestyle modification. However, the issue of gender differences in the effectiveness of control of hypertension is still controversial [44].

The high coverage of the active HT screening program by healthcare workers with a low controlled hypertension and effective coverage (Table 3) means that the effective behavioral modification and treatment procedures need to be improved. Studies showed non-adherence to HT control procedures and lack of healthcare workers were mentioned [46, 47]. We have not explored those two factors in Tibetan context.

This study presents a comprehensive analysis of the coverage and effectiveness of hypertension screening and control at different altitude levels of Tibet.

### Limitations

In a field survey that asks people to participate in the study, it has a high tendency of missing those with severe diseases such as those with CVD complications. Hence, the estimates we reported may be biased towards mildly and moderately severe disease. There were advantages in approaching people in situ at the villages but men working in cities and children studying in schools could be missed. Although we applied post-hoc weights to different age groups and gender by raking procedure, such the adjustment could bring bias in the computation. Also, we could not determine in detail the specific lifestyle modification reported by participants such as daily salt consumption, the number of alcoholic drinks, and the physical activity level. Another limitation in our study is that the study ran under the structure of the healthcare system of the Tibet Government where it is taking care of disease and high-risk persons. Thus, it is unavoidable that we would recruit participants with a high risk of HT and other chronic diseases. It requires further studies to consider the pre-HT, suspected HT cases, and intervention effectiveness, including treatment and CVD risk assessment.

## Conclusion

The study confirmed a high coverage of the hypertension screening program, especially at the low altitude areas, but the effectiveness of the program is improvable. The HT control effectiveness seems to be better among younger females regardless of taken antihypertension medicine or keep a healthy lifestyle or consideration to both. It is possible to run a co-intervention program to improve the adherence to hypertension treatment along with the existing HT screening routines.

## Supplementary Information

**Additional file 1: Supplementary Table 1** Questionnaire and record form for physical measurement.

**Additional file 2: Supplementary Table 2** Characteristics of the participants by screening of hypertension in the past (raw data, *N* = 1636).

## Data Availability

The datasets generated and/or analyzed during the current study are not publicly available due to confidentiality, but data is accessible from the corresponding author on reasonable request.

## Abbreviations

HT: Hypertension
BP: Blood pressure
CVD: Cardiovascular diseases
BMI: Body mass index
SBP: Systolic blood pressure
DBP: Diastolic blood pressure
CI: Confidence interval
