# Supplementary material for: Coverage and effectiveness of hypertension screening in different altitudes of Tibet autonomous region
Source: BMC Public Health. 2021 Jan 6;21:33. doi: 10.1186/s12889-020-09858-0 (PMC7788880; doi:10.1186/s12889-020-09858-0)
Supplement: Supplementary file 1 — Additional file 1: Supplementary Table 1 Questionnaire and record form for physical measurement. [file 12889_2020_9858_MOESM1_ESM.docx]

**Supplementary Table 1** Questionnaire and record form for physical measurement.

| **Part I . Record form for physical measurement** | | | | | | | | | | | | | | | | | | | | | |
| --- | --- | --- | --- | --- | --- | --- | --- | --- | --- | --- | --- | --- | --- | --- | --- | --- | --- | --- | --- | --- | --- |
| Research ID | | |  | | | | | | | | | | | | | | | | | | |
| Items | | | | | | | | | | | | | | Code | | | | | | | |
| Anthropometric measures | | | High (cm) | | | | | Ht | | | | | | [ ][ ][ ] | | | | | | | |
|  |  |  | Weight (kg) | | | | | Wt | | | | | | [ ][ ][ ] | | | | | | | |
|  |  |  | Waist circumference (cm) | | | | | Wc | | | | | | [ ][ ][ ] | | | | | | | |
|  |  |  | Hip circumference (cm) | | | | | Hip | | | | | | [ ][ ][ ] | | | | | | | |
| Research ID | | |  | | | | | | | | | | | | | | | | | | |
| Items | | | | | | | | | | | | | | Code | | | | | | | |
| The measurement result of blood pressure (mmHg) | | | The first time | | | | | SBP1 | | | | | | [ ][ ][ ] | | | | | | | |
|  |  |  |  |  |  |  |  | DBP1 | | | | | | [ ][ ][ ] | | | | | | | |
|  |  |  | The second time | | | | | SBP2 | | | | | | [ ][ ][ ] | | | | | | | |
|  |  |  |  |  |  |  |  | DBP2 | | | | | | [ ][ ][ ] | | | | | | | |
|  |  |  | The third time | | | | | SBP3 | | | | | | [ ][ ][ ] | | | | | | | |
|  |  |  |  |  |  |  |  | DBP3 | | | | | | [ ][ ][ ] | | | | | | | |
| **Part Ⅱ .General information** | | | | | | | | | | | | | | | | | | | | | |
| Research ID | | |  | | | | | | | | | | | | | | | | | | |
| No. | Question Items | | | | | | | | | | | | Code | | | | | | | | |
| G1 | Sex (as observed) ☐ 1 Male ☐ 2 Female | | | | | | | | | | | | Sex | | | | | | [ ] | | |
| G2 | What is your ethnicity?  ☐ 1 Tibetan ☐ 2 Han ☐ 3 Muslim  ☐ 4 Other (specify__________________) | | | | | | | | | | | | Eth | | | | | | [ ] | | |
| G3 | What is your date of birth?  └─┴─┴─┴─┘└─┴─┘└─┴─┘ Year (AD.) mm dd  Don’t Know: 9999  If don’t know, ask age (years). | | | | | | | | | | | | Ybi  Mbi  Dbi  Age | | | | | [ ][ ][ ][ ]  [ ][ ]  [ ][ ]  [ ][ ] | | | |
| G4 | What is your highest level of education you have completed?  ☐ 1 Less than primary school ☐ 2 Primary school  ☐ 3 Secondary school ☐ 4 High school  ☐ 5 Vocational college  ☐ 6 Bachelor degree or higher | | | | | | | | | | | | Edu | | | | | | [ ] | | |
| G5 | What is your marital status?  ☐ 1 Single ☐ 2 Cohabitating ☐ 3 Married  ☐ 4 Separated ☐ 5 Divorced ☐ 6 Widowed | | | | | | | | | | | | Ms | | | | | | [ ] | | |
| G6 | What is your religion?  ☐ 1 Buddhism ☐ 2 Islam  ☐ 3 Christianity ☐ 4 Other(specify________) | | | | | | | | | | | | Rel | | | | | | [ ] | | |
| G7 | What is your main current occupation? (one choice only)  ☐ 1 Civil servant ☐ 2 Private business owner  ☐ 3 Farmer ☐ 4 Herdsman ☐ 5 Laborer  ☐ 6 Unemployed (including retired employee)  ☐ 7 Other (specify_____________________) | | | | | | | | | | | | Occ | | | | | | [ ] | | |
| G8 | How many people, including yourself, live in your household?  └─┴─┘ person (s) | | | | | | | | | | | | Hho | | | | | | [ ][ ] | | |
| G9 | What is your average of monthly household income, before taxes (family) and from all sources?  ☐ 1) Less than RMB2,500 ☐ 2) RMB2,500 ~5,000  ☐ 3) RMB 5,001~7,500 ☐ 4) RMB 7,501~10,000  ☐ 5) More than RMB 10,001 | | | | | | | | | | | | Inc | | | | | | [ ] | | |
| **Part Ⅲ. Utilization of health services** | | | | | | | | | | | | | | | | | | | | | |
| Health Services | | | | | | | | | | | | | | | | | | | | | |
| No. | | Question Items | | | | | | | | | | | | | Code | | | | | | |
| B1 | | What kind of your health insurance?  ☐ 1 FMS: Free Medical Service  ☐ 2 UEBMI: Urban employee basic medical insurance  ☐ 3 URBMI: Urban residents basic medical insurance  ☐ 4NRCMS: New Rural Co-operative Medical System  ☐ 5 CHI: Commercial medical insurance  ☐ 6 Others | | | | | | | | | | | | | B1 | | | | [ ] | | |
| B2 | | For general disease, usually you preferred which type hospital for seeking treatment?  ☐ 1 Village health center  ☐ 2 Township health center  ☐ 3 County hospital  ☐ 4 Municipal hospital  ☐ 5 Provincial hospital  ☐ 6 Other: . | | | | | | | | | | | | | B2 | | | | [ ] | | |
| B3 | | How many kilometres from your home to the nearest medical institution? km | | | | | | | | | | | | | B3 | | | | [ ] [ ] | | |
| B4 | | How many minutes from your home to the nearest medical institutions? ( Fastest way get to the destination by walk or other transportation) minutes | | | | | | | | | | | | | B4 | | | | [ ] [ ] | | |
| B5 | | Usually you preferred which type health service for treatment?  ☐ 1 Traditional Tibetan medicine  ☐ 2 Western medicine  ☐ 3 Traditional Chinese medicine | | | | | | | | | | | | | B5 | | | | [ ] | | |
| B6 | | Give one most important reason why you preferred that health service?  ☐ 1 Good service attitude  ☐ 2 Because use same language easy understand  ☐ 3 Good medical condition  ☐ 4 Convenient treatment  ☐ 5 other reason (specify__________________) | | | | | | | | | | | | | B6 | | | | [ ] | | |
| Management and education received from local health service centre | | | | | | | | | | | | | | | | | | | | | |
| M1 | | Is there family history of hypertension?  ☐ 1 Yes ☐ 2 No | | | | | | | | | | | | | M1 | | | | [ ] | | |
| M2 | | Have been you screened for diagnosed high blood or (hypertension) by the health worker during annals physical examination or generally hypertensive intervention programs?  ☐ 1 Yes, I had been screened and diagnosed HT  ☐ 2 Yes, I had been screened, but the result of measure is normal *(Skip to T1)*  ☐ 3 No, I never attend those programs and, I also don’t know about my blood pressure status. *(Skip to M6 )* | | | | | | | | | | | | | M 2 | | | | [ ] | | |
| M3 | | How long have you been diagnosed with hypertension?  ☐ 1) < 1 month ☐ 2) 1 month -11month  ☐ 3) 1-9.9 years ☐ 4) ≥ 10 years | | | | | | | | | | | | | M3 | | | | [ ] | | |
| M4 | | During the past two weeks, have you been treated for raised blood pressure with drugs (medication) prescribed?  ☐ 1 Yes ☐ 2 No (Skip to M5 ) | | | | | | | | | | | | | M4 | | | | [ ] | | |
| M4.1 | | If the answer is “yes”, you used which kind of medicine to prescribe?  ☐ 1 Traditional Tibetan medicine  ☐ 2 Western medicine  ☐ 3 Traditional Chinese medicine | | | | | | | | | | | | | M4.1 | | | | [ ] | | |
| M5 | | In the past 12 months, How often do you see your doctor for blood pressure check-ups?  ☐ 1 Monthly ☐ 2 Every 2 – 5 months  ☐ 3 Every 6 months ☐ 4 More than 6 months  ☐ 5 Not in the past 12 month | | | | | | | | | | | | | M5 | | | | [ ] | | |
| M6 | | Do you have any underlying disease?  ☐ 1 Yes ☐ 2 No  *(Skip to T1)* | | | | | | | | | | | | | M6 | | | | [ ] | | |
| M7 | | Have you ever suffered from any of the following conditions? | | | | | | | | | | 1=Yes | | | | 2 =No | | | | | Code |
| M7.1 | | Diabetes Mellitus | | | | | | | | | | ☐ | | | | ☐ | | | | | [ ] |
| M7.2 | | High Cholesterol | | | | | | | | | | ☐ | | | | ☐ | | | | | [ ] |
| M7.3 | | Angina | | | | | | | | | | ☐ | | | | ☐ | | | | | [ ] |
| M7.4 | | Stroke | | | | | | | | | | ☐ | | | | ☐ | | | | | [ ] |
| M7.5 | | Intermittent claudication | | | | | | | | | | ☐ | | | | ☐ | | | | | [ ] |
| M7.6 | | Poor vision | | | | | | | | | | ☐ | | | | ☐ | | | | | [ ] |
| M7.7 | | Kidney disease | | | | | | | | | | ☐ | | | | ☐ | | | | | [ ] |
| M7.8 | | Not sure | | | | | | | | | | ☐ | | | | ☐ | | | | | [ ] |
| The result of follow up service records for patient with hypertension in past | | | | | | | | | | | | | | | | | | | | | |
| M8 | | Follow up data | | Follow up way | | Code | SBP (mmHg) | | | | | | | | | | DBP (mmHg) | | | | |
| M8.1 | | /Y /M /D | | ☐ 1 Outpatient  ☐ 2 Household  ☐ 3 Telephone | | [ ] | [ ][ ][ ] | | | | | | | | | | [ ][ ][ ] | | | | |
| M8.2 | | /Y /M /D | | ☐ 1 Outpatient  ☐ 2 Household  ☐ 3 Telephone | | [ ] | [ ][ ][ ] | | | | | | | | | | [ ][ ][ ] | | | | |
| M8.3 | | /Y /M /D | | ☐ 1 Outpatient  ☐ 2 Household  ☐ 3 Telephone | | [ ] | [ ][ ][ ] | | | | | | | | | | [ ][ ][ ] | | | | |
| M8.4 | | /Y /M /D | | ☐ 1 Outpatient  ☐ 2 Household  ☐ 3 Telephone | | [ ] | [ ][ ][ ] | | | | | | | | | | [ ][ ][ ] | | | | |
| Characteristic of taking anti-hypertensive medication | | | | | | | | | | | | | | | | | | | | | |
| U1 | | Are you taking anti-hypertension medication pills to control your HT?  ☐ 1 Yes ☐ 2 No (Skip to U20) | | | | | U1 | | | | | | | | | | [ ] | | | | |
| U2 | | Which kind of anti-hypertensive medicine are you taking? | | | | | 1=Yes | | | | | | | | 2 =No | | | | | | Code |
| U2.1 | | Traditional Tibetan medicine | | | | | ☐ | | | | | | | | ☐ | | | | | | [ ] |
| U2.2 | | Western medicine | | | | |  | | | | | | | |  | | | | | |  |
| U2.3 | | Traditional Chinese medicine | | | | | ☐ | | | | | | | | ☐ | | | | | | [ ] |
| U2.4 | | Others (If “Yes”, the specify is ) | | | | | ☐ | | | | | | | | ☐ | | | | | | [ ] |
| U3 | | Why did you choose this way of anti-hypertension medicine? | | | | | 1 = Agree | | | | | | | | 2 = Disagree | | | | | | Code |
| U3.1 | | Not especially reason just comply doctor’s advice | | | | | ☐ | | | | | | | | ☐ | | | | | | [ ] |
| U3.2 | | I like us culture, I believe that medicine can help me cure | | | | | ☐ | | | | | | | | ☐ | | | | | | [ ] |
| U3.3 | | Can’t afford expensive medicine only select it. | | | | | ☐ | | | | | | | | ☐ | | | | | | [ ] |
| U3.4 | | Blood pressure could be effectively controlled by currently this drug. | | | | | ☐ | | | | | | | | ☐ | | | | | | [ ] |
| U3.5 | | Before anti-hypertension medicine was not effective or cause side-effects so want to change present drugs. | | | | | ☐ | | | | | | | | ☐ | | | | | | [ ] |
| U3.6 | | Recommended by friends or relatives around | | | | | ☐ | | | | | | | | ☐ | | | | | | [ ] |
| U3.7 | | Other reason | | | | | ☐ | | | | | | | | ☐ | | | | | | [ ] |
| U4 | | The village health worker introduced the important of adherence to medication?  ☐ 1 Yes ☐ 2 No | | | | | | | | | | | | | U4 | | | | | | [ ] |
| U5 | | Have you ever met health worker to get any advice regarding how to take medicines correctly?  ☐ 1 Yes ☐ 2 No | | | | | | | | | | | | | U5 | | | | | | [ ] |
| U6 | | How long have you been taking your current anti-hypertension medicine? (record rough time)  └─┴─┘years | | | | | | | | | | | | | U6 | | | | | | [ ] |
| U7 | | Who **mostly** helps you taking your anti-hypertension? (one choice for type of medication)  ☐ 1 Myself ☐ 2 Family member  ☐ 3 Friend ☐ 4 Others(specify) ______­­­­­­­­­­­_ | | | | | | | | | | | | | U7 | | | | | | [ ] |
| U8 | | Have you ever had any adverse effects of anti-hypertension medication?  ☐ 1 Yes ☐ 2 No (Skip to U12）  ☐ 9 Not sure / Don’t know (Skip to U12) | | | | | | | | | | | | | U8 | | | | | | [ ] |
| U9 | | What adverse effects did you have? | | | 1 = Yes, it is  (unprompted) | | | | 2 = Yes | | | | | | 3 = No | | | | | | Code |
| U9.1 | | Palpitation | | | ☐ | | | | ☐ | | | | | | ☐ | | | | | | [ ] |
| U9.2 | | Fatigue | | | ☐ | | | | ☐ | | | | | | ☐ | | | | | | [ ] |
| U9.3 | | Dizziness | | | ☐ | | | | ☐ | | | | | | ☐ | | | | | | [ ] |
| U9.4 | | Abdominal discomfort | | | ☐ | | | | ☐ | | | | | | ☐ | | | | | | [ ] |
| U9.5 | | Diarrhea | | | ☐ | | | | ☐ | | | | | | ☐ | | | | | | [ ] |
| U9.6 | | Rash | | | ☐ | | | | ☐ | | | | | | ☐ | | | | | | [ ] |
| U9.7 | | Other | | | ☐ | | | | ☐ | | | | | | ☐ | | | | | | [ ] |
| U10 | | About those adverse effects, are you had talked to health worker?  ☐ 1 Yes ☐ 2 No *(Skip to U12*) | | | | | | | | | | | | | U10 | | | | | | [ ] |
| U11 | | The health worker give you any advised for your adverse effect?  ☐ 1 Yes (let me change drug or go to a superior hospital and further physical examination et al.)  ☐ 2 No | | | | | | | | | | | | | U11 | | | | | | [ ] |
| **Part Ⅳ. Medication Adherence Scale for Hypertensive Patients**  You indicated that you are taking medication for your high blood pressure. Individuals have identified several issues regarding their medication-taking behaviour and we are interested in your experiences. There is no right or wrong answer. Please answer each question based on your personal experience with your hypertension medication. | | | | | | | | | | | | | | | | | | | | | |
| No. | | Question items | | | | | No=0 | | | | | | | | Yes=1 | | | | | Code | |
| U12 | | Do you sometimes forget to take your blood pressure pills? | | | | | ☐ | | | | | | | | ☐ | | | | | [ ] | |
| U13 | | People sometimes miss taking their medications for reasons other than forgetting. Thinking over the past two weeks, were there any days when you did not take your blood pressure medicine? | | | | | ☐ | | | | | | | | ☐ | | | | | [ ] | |
| U14 | | Have you ever cut back or stopped taking your medication without telling your doctor because you felt worse when you took it? | | | | | ☐ | | | | | | | | ☐ | | | | | [ ] | |
| U15 | | When you travel or leave home, do you sometimes forget to bring along your blood pressure medication? | | | | | ☐ | | | | | | | | ☐ | | | | | [ ] | |
| U16 | | Did you take your blood pressure medicine yesterday? | | | | | ☐ | | | | | | | | ☐ | | | | | [ ] | |
| U17 | | When you feel like your blood pressure is under control, do you sometimes stop taking your medicine? | | | | | ☐ | | | | | | | | ☐ | | | | | [ ] | |
| U18 | | Taking medication every day is a real inconvenience for some people. Do you ever feel hassled about sticking to your blood pressure treatment plan? | | | | | ☐ | | | | | | | | ☐ | | | | | [ ] | |
| U19 | | How often do you have difficulty remembering to take all your medications? (Please circle the correct number) [ ]  Never/Rarely ☐ 4 Once in a while ☐ 3  Sometimes ☐ 2 Usually ☐ 1 All the time ☐ 0 | | | | | | | | | | | | | | | | | | [ ] | |
| U20 | | The reason for didn’t taken medication : | | | | | No=0 | | | | | | | | Yes=1 | | | | | Code | |
| U20.1 | | Don’t know where can receive treatment | | | | | ☐ | | | | | | | | ☐ | | | | | [ ] | |
| U20.2 | | Can’t afford | | | | | ☐ | | | | | | | | ☐ | | | | | [ ] | |
| U20.3 | | Don’t believe the disease can be cured | | | | | ☐ | | | | | | | | ☐ | | | | | [ ] | |
| U20.4 | | Without health facility in the local area | | | | | ☐ | | | | | | | | ☐ | | | | | [ ] | |
| U20.5 | | Transportation inaccessible | | | | | ☐ | | | | | | | | ☐ | | | | | [ ] | |
| U20.6 | | Transportation inaccessible | | | | | ☐ | | | | | | | | ☐ | | | | | [ ] | |
| U20.7 | | Other reasons | | | | | ☐ | | | | | | | | ☐ | | | | | [ ] | |
| **Part Ⅴ. Life behaviors questionnaire** | | | | | | | | | | | | | | | | | | | | | |
| Tobacco use | | | | | | | | | | | | | | | | | | | | | |
| No. | | Items | | | | | | | | | | | | | Code | | | | | | |
| T1 | | Have you ever smoked cigarettes, such as cigarettes, cigars or pipes?  ☐ 1 Yes ☐ 2 No (Skip to A1) | | | | | | | | | | | | | T1 | | | | | [ ] | |
| T2 | | How old were you when you first started smoking?  year. | | | | | | | | | | | | | T2 | | | | | [ ] | |
| T3 | | How long did you smoke? years. | | | | | | | | | | | | | T3 | | | | | [ ] | |
| T4 | | On average how many cigarettes are you/have you been taking in a day?  ☐ 1) 1-9 ☐ 2) 10-19 ☐ 3) 20 or >20 | | | | | | | | | | | | | T4 | | | | | [ ] | |
| T5 | | Health worker had a suggestion you smoking cessation?  ☐ 1 Yes ☐ 2 No | | | | | | | | | | | | | T5 | | | | | [ ] | |
| T6 | | Do you currently smoke tobacco products daily?  ☐ 1 Yes (Skip to A1) ☐ 2 No | | | | | | | | | | | | | T6 | | | | | [ ] | |
| T6.1 | | When did you quit smoking? Before: years | | | | | | | | | | | | | T6.1 | | | | | [ ] | |
| Alcohol consumption | | | | | | | | | | | | | | | | | | | | | |
| A1 | | Have you ever drink alcohol, (including any kind of alcohol, such as Chaung or barley wine?)  ☐ 1 Yes ☐ 2 No (Skip to D1) | | | | | | | | | | | | | A1 | | | | | [ ] | |
| A2 | | Usually which brand of alcohol you like to drink? (SHOW CARD) | | | | | | | | | No=0 | | | | Yes=1 | | | | | Code | |
| A2.1 | | Bear | | | | | | | | | ☐ | | | | ☐ | | | | | [ ] | |
| A2.2 | | Highland barley wine or Chuang | | | | | | | | | ☐ | | | | ☐ | | | | | [ ] | |
| A2.3 | | White wine | | | | | | | | | ☐ | | | | ☐ | | | | | [ ] | |
| A2.4 | | Rice wine | | | | | | | | | ☐ | | | | ☐ | | | | | [ ] | |
| A2.5 | | Grape wine | | | | | | | | | ☐ | | | | ☐ | | | | | [ ] | |
| A2.6 | | Other brand alcohol | | | | | | | | | ☐ | | | | ☐ | | | | | [ ] | |
| A3 | | How often did you drink beer, wine, grape (including all kind of alcohol) or any other alcoholic beverage, even in small amounts, for example, a glass of beer, in the past 12 months? (SHOW CARD)  ☐ 1 Every day ☐ 2 5-6 times a week  ☐ 3 3-4 times a week ☐ 4 1-2 times a week  ☐ 5 2-3 times a month ☐ 6 One a month  ☐ 7 < 6times /year  ☐ 8 I did not drink last 12 month, but drank earlier  ☐ 9 I never drank in my life | | | | | | | | A3.1 Bear | | | | | | | | | | [ ] | |
|  |  |  |  |  |  |  |  |  |  | A3.2 Chuang | | | | | | | | | | [ ] | |
|  |  |  |  |  |  |  |  |  |  | A3.3 White wine | | | | | | | | | | [ ] | |
|  |  |  |  |  |  |  |  |  |  | A3.4 Rice wine | | | | | | | | | | [ ] | |
|  |  |  |  |  |  |  |  |  |  | A3.5 Grape wine | | | | | | | | | | [ ] | |
|  |  |  |  |  |  |  |  |  |  | A3.6Other brand | | | | | | | | | | [ ] | |
| A4 | | The village health worker had a suggestion to you abstinence?  ☐ 1 Yes ☐ 2 No | | | | | | | | | | | | | A4 | | | | | [ ] | |
| Dietary characteristics | | | | | | | | | | | | | | | | | | | | | |
| D1 | | Is your hypertension controlled with diet?  ☐ 1 Yes ☐ 2 No | | | | | | | | | | | | | D1 | | | | | [ ] | |
| D2 | | Have you ever received diet recommendation from health worker to keep healthy lifestyle (such as control hypertension)?  ☐ 1 Yes ☐ 2 No  *( Skip to D5)* | | | | | | | | | | | | | D2 | | | | | [ ] | |
| D3 | | How often do you consume fruits and vegetables of all kinds (fresh, canned, frozen, cooked, raw and juices)?  ☐ 1) <1time /day ☐ 2) 1 time/day  ☐ 3) 2 times/day ☐ 4) >3times/day | | | | | | | | | | | | | D3 | | | | | [ ] | |
| D4 | | Among those recommendations diet, which kind of food you cannot follow or find difficult to follow: | | | | | | | | | | | | | Code | | | | | | |
| D4.1 | | Avoid sweet food  ☐ 1 Yes ☐ 2 No ☐ 9 Don’t know | | | | | | | | | | | | | D4.1 | | | | | [ ] | |
| D4.2 | | Avoid salty food  ☐ 1 Yes ☐ 2 No ☐ 9 Don’t know | | | | | | | | | | | | | D4.2 | | | | | [ ] | |
| D4.3 | | Avoid high fat food  ☐ 1 Yes ☐ 2 No ☐ 9 Don’t know | | | | | | | | | | | | | D4.3 | | | | | [ ] | |
| D4.4 | | Eat more vegetable and fruit  ☐ 1 Yes ☐ 2 No ☐ 9 Don’t know | | | | | | | | | | | | | D4.4 | | | | | [ ] | |
| D4.5 | | Eat more fish and egg  ☐ 1 Yes ☐ 2 No ☐ 9 Don’t know | | | | | | | | | | | | | D4.5 | | | | | [ ] | |
| D5 | | The village health worker had suggestion to you intake low salt?  ☐ 1 Yes ☐ 2 No | | | | | | | | | | | | | D5 | | | | | [ ] | |
| D6 | | Are you know the maximum daily amount of salt recommended for adults?  ☐ 1 Yes ☐ 2 No *( skip to D7)* | | | | | | | | | | | | | D6 | | | | | [ ] | |
| D6.1 | | In the recommended the maximum daily salt intake  .g/day | | | | | | | | | | | | | D6.1 | | | | | [ ] | |
| D7 | | Are you concerned about the amount of salt/sodium in the diet?  ☐ 1 Yes ☐ 2 No ☐ 3 Nature | | | | | | | | | | | | | D7 | | | | | [ ] | |
| D8 | | Are you agreed the behavior of reduce sodium intake is definitely important to your health?  ☐ 1 Agree ☐ 2 Disagree ☐ 3 Nature | | | | | | | | | | | | | D8 | | | | | [ ] | |
| D9 | | Are you have been try to buy “no added salt” foods?  ☐ 1 Often ☐ 2 Sometimes ☐ 3 Never try | | | | | | | | | | | | | D9 | | | | | [ ] | |
| D10 | | Are you think you are overweight?  ☐ 1 Yes ☐ 2 No, my BMI is normal *( skip to D12)*  ☐ 9 I don’t know *( skip to D12)* | | | | | | | | | | | | | D10 | | | | | [ ] | |
| D11 | | Local health worker had a suggestion to you control weight or lose weight?  ☐ 1 Yes ☐ 2 No | | | | | | | | | | | | | D11 | | | | | [ ] | |
| D12 | | Village health worker had suggestion to you keeping exercise?  ☐ 1 Yes ☐ 2 No | | | | | | | | | | | | | D12 | | | | | [ ] | |
| Physical activity | | | | | | | | | | | | | | | | | | | | | |
| Next, I am going to ask you about the time you spend doing different types of physical activity in the LAST SEVEN DAYS. Think first about the time you spend DOING WORK. Work is anything you have to do such as paid or unpaid work, study/training, household chores, harvesting food/crops, farming or hunting for food…,  Follows are examples of **WORKS.**  Housework: mopping, sweeping, vacuuming  Gardening: planting, raking, weeding rubber tapping  Other activities: mowing lawn, farming (with tractor), power mower, carpentry, carry light load, washing car, grazing, the turning  In answering the following questions 'vigorous-intensity activities' are activities that require hard physical effort and make a LARGE increases in breathing or heart rate(Items P2 – P2b), 'moderate-intensity activities' are activities that require moderate physical effort and makes a SMALL increase in your breathing or heart rate(Items P1 – P1b). | | | | | | | | | | | | | | | | | | | | | |
| No. | | Items | | | | | | | | | | | | | Code | | | | | | |
| Active at work | | | | | | | | | | | | | | | | | | | | | |
| P1 | | Does your **WORK** involved moderate-intensity activities that mad a **SMALL** increase in your breathing or heart rate continuously for at least 10 minutes at a time during **LAST SEVEN DAYS**?  ☐ 1 Yes ☐ 2 No  *(skip to P2)* | | | | | | | | | | | | | P1 | | | | | [ ] | |
| P1a | | During the **LAST SEVEN DAYS,** on how many days did you do a moderate physical activity as part of your work continuously for at least 10 minutes at a time? (If answer is 0 day, skip to P2) .day(s). | | | | | | | | | | | | | P1a | | | | | [ ] | |
| P1b | | How much time did you spend on average doing moderate physical activities at work per day?  └─┴─┴─┘min/day | | | | | | | | | | | | | P1b | | | | | [ ] | |
| P2 | | Has does your **WORK** involved vigorous-intensity activities that made LARGE increase in your breathing or heart rate continuously for at least 10 minutes at a time during **LAST SEVEN DAYS**?  ☐ 1 Yes ☐ 2 No *(skip to P3 )* | | | | | | | | | | | | | P2 | | | | | [ ] | |
| P2a | | During the **LAST SEVEN DAYS**, on how many days did you do a vigorous physical activity as part of your **WORK** continuously for at least 10 minutes at a time? (If answer is 0 day, *skip to P3*)  .day(s). | | | | | | | | | | | | | P2a | | | | | [ ] | |
| P2b | | How much time did you spend on average doing vigorous physical activities at work per day?  └─┴─┴─┘min/day | | | | | | | | | | | | | P2b | | | | | [ ] | |
| Now I would like to ask you about moderate exercise and sports activity (P3 –P3b)  Sport or exercise activity at a moderate level is any activities that are somewhat hard and makes a **SMALL** increase in breath or heart rate.  Example are: Fast walking, low impact aerobics, volleyball, Prayer kowtow, yoga, Tai chi, qigong, weight lifting, dancing, horse riding | | | | | | | | | | | | | | | | | | | | | |
| No. | | Question Items | | | | | | | | | | | | | Code | | | | | | |
| Recreational activities | | | | | | | | | | | | | | | | | | | | | |
| P3 | | Have you done any exercise or sports activities that made a **SMALL** increase in your breathing or heart rate continuously for at least 10 minutes at a time during **LAST SEVEN DAYS**?  ☐ 1 Yes ☐ 2 No *(skip to P4)* | | | | | | | | | | | | | P3 | | | | | [ ] | |
| P3a | | During the **LAST SEVEN DAYS**, on how many days did you do a moderate exercise or sports activity continuously for at least 10 minutes at a time? (If answer is 0 day, skip to P4)  .day(s). | | | | | | | | | | | | | P3a | | | | | [ ] | |
| P3b | | On those days, how much time per day did you spend on average doing moderate exercise or sports activities?  └─┴─┴─┘min/day | | | | | | | | | | | | | P3b | | | | | [ ] | |
| Now I would like to ask you about vigorous exercise and sport activity(P4 – P4b)  Sport or exercise activity at vigorous  level is any activities that is somewhat hard and makes a LARGE increase in breath or heart rate or you feel like you do when you run or jog.  Examples are: jogging, running, high impact aerobics (Jazzercise), swimming laps, jumping rope, basketball, tennis, fast bicycling (more than 20km /hour), Judo, karate, kickboxing. | | | | | | | | | | | | | | | | | | | | | |
| No. | | Items | | | | | | | | | | | | | Code | | | | | | |
| P4 | | Have you done any exercise and sport activities that made a **LARGE** increase in your breathing or heart rate during your last **SEVEN DAYS**?  ☐ 1 Yes ☐ 2 No *(skip to P5)* | | | | | | | | | | | | | P4 | | | | | [ ] | |
| P4a | | During the **LAST SEVEN DAYS**, on how many days did you do a vigorous exercise/sport activity continuously for at least 10 minutes at a time? (If answer is 0 days, *skip to P5*)  .day(s). | | | | | | | | | | | | | P4a | | | | | [ ] | |
| P4b | | On those days, how much time per day did you spend on average doing vigorous exercise/sport activities?  └─┴─┴─┘min/day | | | | | | | | | | | | | P4b | | | | | [ ] | |
| Now I would like to ask (P5 – P5b) you about the usual way you travel to and from places. For example to work, for shopping, to market, to place of worship  (NOTES: excluded activities like walking or pedal-bicycle that you have already mentioned as exercise/sport activities ) | | | | | | | | | | | | | | | | | | | | | |
| P5 | | Have you walked or used a bicycle (pedal cycle) for at least 10 minutes continuously to get to and from places **DURING THE LASTSEVEN DAYS**?  ☐ 1 Yes ☐ 2 No (skip to P6) | | | | | | | | | | | | | P5 | | | | | [ ] | |
| P5a | | During the **LAST 7 DAYS**, on how many days did you walk or bicycle for at least 10 minutes continuously to get to and from places? (If answer is 0 day, skip toP6)  day(s). | | | | | | | | | | | | | P5a | | | | | [ ] | |
| P5b | | How much time did you spend walking or bicycling for travel per day?  └─┴─┴─┘min/day | | | | | | | | | | | | | P5b | | | | | [ ] | |
| P6 | | Compared with your Usual Physical Activity over the **LAST 3 MONTHS**, was the **LAST SEVEN** **DAYS’** activity:  ☐ 1 More   ☐ 2 Less ☐ 3 Same | | | | | | | | | | | | | p6 | | | | | [ ] | |
| P7 | | Over the last 12 months, have you ever been given advice by a health worker (doctor, nurse,….) about physical activity levels proper for your health condition (such as HT)?  ☐ 1 Yes ☐ 2 No *(skip to P9)* | | | | | | | | | | | | | P7 | | | | | [ ] | |
| P7.1 | | Can you follow the advice?  ☐ 1 Yes, always *(skip to P9)*  ☐ 2 Yes, sometimes *(skip to P9)*  ☐ 3 Rarely ☐ 4 Not at all | | | | | | | | | | | | | P7.1 | | | | | [ ] | |
| P8 | | Which reasons that make you cannot follow the physical activity advice among following reason? | | | | | | | | | | | | | | | | | | | |
| P8.1 | | The advice or guideline is not clear enough?  ☐ 1 Yes ☐ 2 No | | | | | | | | | | | | | P8.1 | | | | | [ ] | |
| P8.2 | | The advice or guideline is not feasible for you? (such as (time, equipment, occupation, etc.)  ☐ 1 Yes ☐ 2 No | | | | | | | | | | | | | P8.2 | | | | | [ ] | |
| P8.3 | | You are awareness the benefits of exercise, but you are willingness was not enough strong?  ☐ 1 Yes ☐ 2 No | | | | | | | | | | | | | P8.3 | | | | | [ ] | |
| P8.4 | | Other reason  ☐ 1 Yes ☐ 2 No | | | | | | | | | | | | | P8.4 | | | | | [ ] | |
| The following question is about sitting or reclining at work, at home, getting to and from places, or with friends including time spent [sitting at a desk, sitting with friends, travelling car, bus, train, reading, playing cards or watching televise on], but do not include time spent sleeping. | | | | | | | | | | | | | | | | | | | | | |
| P9 | | How much time do you usually spend sitting or reclining on a typical day?  └─┴─┘Hours | | | | | | | | | | | | | P9 | | | | | [ ] | |
| **Part VI. Hypertension Knowledge-Level Scale (HK-LS)** | | | | | | | | | | | | | | | | | | | | | |
