# Supplementary material for: Coverage and effectiveness of hypertension screening in different altitudes of Tibet autonomous region
Source: BMC Public Health. 2021 Jan 6;21:33. doi: 10.1186/s12889-020-09858-0 (PMC7788880; doi:10.1186/s12889-020-09858-0)
Supplement: Supplementary file 2 — Additional file 2: Supplementary Table 2 Characteristics of the participants by screening of hypertension in the past (raw data, N = 1636). [file 12889_2020_9858_MOESM2_ESM.docx]

**Supplementary Table 2** Characteristics of the participants by screening of hypertension in the past (raw data, N=1,636).

| Characteristic | Ever screened for hypertension in the past | | | | | p- value |
| --- | --- | --- | --- | --- | --- | --- |
|  | Screened | | | | Not screened  n (%) |  |
|  | Total  n (%) | Diagnosed  HT, n (%) | Not diagnosed HT, n (%) | p - value |  |  |
| Total (%) | 1,539 (94.1) | 576 (37.4) | 963 (62.6) |  | 97 (5.9) |  |
| Gender |  |  |  | < 0.001 |  | 0.390 |
| Male | 575 (93.3) | 326 (56.8) | 249 (43.2) |  | 41 (6.7) |  |
| Female | 964 (94.5) | 637 (66.1) | 327 (33.9) |  | 56 (5.5) |  |
| Age group |  |  |  | < 0.001 |  | 0.003 |
| 18 - 39 | 335 (98.0) | 35 (10.4) | 300 (89.6) |  | 7 (2.0) |  |
| 40 - 59 | 902 (93.1) | 325 (36.0) | 577 (64.0) |  | 67 (6.9) |  |
| 60 + | 302 (92.9) | 216 (71.5) | 86 (28.5) |  | 23 (7.1) |  |
| Marital status |  |  |  | < 0.001 |  | 0.001 |
| Single/separated | 241 (98.8) | 143 (59.3) | 98 (10.2) |  | 3 (1.2) |  |
| Married | 1298 (93.2) | 433 (33.4) | 865 (89.8) |  | 94 (6.8) |  |
| Education |  |  |  |  |  | 0.225 |
| None | 835 (94.2) | 338 (40.5) | 497 (59.5) | 0.003 | 51 (5.8) |  |
| Primary school | 615 (93.3) | 217 (35.3) | 398 (64.7) |  | 44 (6.7) |  |
| Middle school and above | 89 (97.8) | 21 (23.6) | 68 (76.4) |  | 2 (2.2) |  |
| Occupation |  |  |  | 0.102 |  | 0.036 |
| Agricultural | 1393 (94.6) | 512 (36.8) | 881 (63.2) |  | 80 (5.4) |  |
| Herdsman | 99 (89.2) | 47 (47.5) | 52 (52.5) |  | 12 (10.8) |  |
| Other | 47 (90.4) | 17 (36.2) | 30 (63.8) |  | 5 (9.6) |  |
| Household monthly income (CNY) | |  |  | 0.296 |  | 0.763 |
| ≤ 2500 | 1361 (93.9) | 518 (38.1) | 843 (61.9) |  | 88 (6.1) |  |
| 2501- 5000 | 152 (95.0) | 51 (33.6) | 101 (66.4) |  | 8 (5.0) |  |
| ≥ 5001 | 26 (96.3) | 7 (26.9) | 19 (73.1) |  | 1 (3.7) |  |
| Tobacco use |  |  |  | 0.603 |  | 0.364 |
| Yes | 388 (93.0) | 150 (38.7) | 238 (61.3) |  | 29 (7.0) |  |
| No | 1151 (94.4) | 426 (37.0) | 725 (63.0) |  | 68 (5.6) |  |
| Alcohol use |  |  |  | 0.066 |  | 0.255 |
| Yes | 303 (95.6) | 99 (32.7) | 204 (67.3) |  | 14 (4.4) |  |
| No | 1236 (93.7) | 477 (38.6) | 759 (61.4) |  | 83 (6.3) |  |
| BMI (kg/m^2^) |  |  |  | < 0.001 |  | 0.011 |
| ≤ 23.9 (Normal) | 849 (95.4) | 255 (30.0) | 594 (70.0) |  | 41 (4.6) |  |
| 24 - 27.9 (Overweight) | 464 (93.5) | 200 (43.1) | 264 (56.9) |  | 32 (6.5) |  |
| ≥ 28.0 (Obese) | 226 (90.4) | 121 (53.5) | 105 (46.5) |  | 24 (9.6) |  |
| Altitude level (m) |  |  |  | < 0.001 |  | < 0.001 |
| Low (2500 - 3499) | 530 (97.2) | 264 (49.8) | 266 (50.2) |  | 15 (2.8) |  |
| Middle (3500 - 4399) | 513 (94.1) | 180 (35.1) | 333 (64.9) |  | 32 (5.9) |  |
| High (4400 - 5300) | 496 (90.8) | 132 (26.6) | 364 (73.4) |  | 50 (9.2) |  |
| SBP (mean+ s.d.) | 132.5 + 24.0 | 150.9 + 24.5 | 121.4 + 15.3 | - | 140.3 + 20.2 | - |
| DBP (mean+ s.d.) | 86.0 + 14.4 | 96.2 + 14.1 | 79.8 + 10.5 |  | 91.6 + 11.9 |  |

* CNY: Chinese yuan
